# Fiber-type traps: revisiting common misconceptions about skeletal muscle fiber types with application to motor control, biomechanics, physiology, and biology

**DOI:** 10.1152/japplphysiol.00337.2023

**Published:** 2023-11-23

**Authors:** Silvia S. Blemker, Susan V. Brooks, Karyn A. Esser, Katherine R. Saul

**Affiliations:** ^1^Department of Biomedical Engineering, University of Virginia, Charlottesville, Virginia, United States; ^2^Department of Molecular and Integrative Physiology, University of Michigan, Ann Arbor, Michigan, United States; ^3^Department of Physiology and Aging, University of Florida, Gainesville, Florida, United States; ^4^Department of Mechanical and Aerospace Engineering, North Carolina State University, Raleigh, North Carolina, United States

**Keywords:** fiber type, myosin, skeletal muscle

## Abstract

Skeletal muscle is a highly complex tissue that is studied by scientists from a wide spectrum of disciplines, including motor control, biomechanics, exercise science, physiology, cell biology, genetics, regenerative medicine, orthopedics, and engineering. Although this diversity in perspectives has led to many important discoveries, historically, there has been limited overlap in discussions across fields. This has led to misconceptions and oversimplifications about muscle biology that can create confusion and potentially slow scientific progress across fields. The purpose of this synthesis paper is to bring together research perspectives across multiple muscle fields to identify common assumptions related to muscle fiber type that are points of concern to clarify. These assumptions include *1*) classification by myosin isoform and fiber oxidative capacity is equivalent, *2*) fiber cross-sectional area (CSA) is a surrogate marker for myosin isoform or oxidative capacity, and *3*) muscle force-generating capacity can be inferred from myosin isoform. We address these three fiber-type traps and provide some context for how these misunderstandings can and do impact experimental design, computational modeling, and interpretations of findings, from the perspective of a range of fields. We stress the dangers of generalizing findings about “muscle fiber types” among muscles or across species or sex, and we note the importance for precise use of common terminology across the muscle fields.

## Graphical abstract

**Figure:**
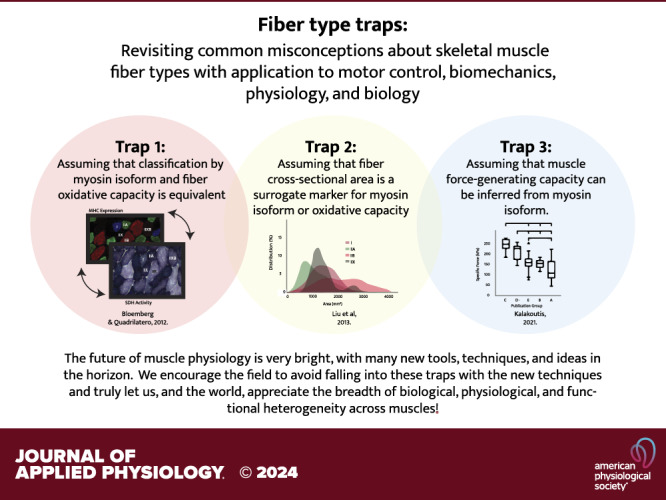

## INTRODUCTION

One of the most well-studied and fundamental characteristics of mammalian skeletal muscle is the speed of muscle shortening. Maximum shortening velocity varies across fibers, leading to the classification of muscle fibers (i.e., cells) into various types. Over the past 100 years, and as distinct fiber classifications were identified and defined, researchers termed these categories “fiber type” and have linked fiber-type phenotype or changes to disease or athletic performance. Over time, associations among myosin isoforms and other structural and functional properties, including metabolism and fatiguability, fiber size, and force generation were reported. Although descriptions of these associations became more common, research ranging from molecular adaptations to muscle mechanics to multiscale modeling has expanded both in breadth and depth. This expansion of research has included new technical approaches and tools, leading to large volumes of data relating to “fiber types”; however, as a muscle research field, we have not stepped back to look critically at when and how fiber-type classifications and associations were established and ask which, if any, of the accepted generalized associations are supported by current research. This is the focus of our discussion and the current perspective piece.

We brought our research experience from different parts of the skeletal muscle research field to discuss three common fiber-type assumptions or potential “traps”. These assumptions include: *1*) classification by myosin isoform and fiber oxidative capacity is equivalent, *2*) fiber cross-sectional area (CSA) is a surrogate marker for myosin isoform or oxidative capacity, and *3*) muscle force-generating capacity can be inferred from myosin isoform. In all three cases we found that these common assumptions are not universally valid when one considers updated understanding about muscle fibers across all the different muscles in the body as well as the variation among species. We recently had an opportunity to talk to a large audience of skeletal muscle researchers at the 2023 Advances in Skeletal Muscle Biology meeting. We presented these three traps and found that, even among researchers with a specialty in skeletal muscle science, there was a still diversity of perspectives on these fiber-type assumptions. There was also enthusiasm for the importance of clarifying how these traps came to be and what pitfalls to look out for when performing, interpreting, and reviewing research.

In this piece, we provide a brief perspective on the history that led to each trap and then discuss the data demonstrating that these issues are more complex than is frequently conveyed. We are not questioning the validity or utility of classifying muscle fibers according to their underlying features; rather our hope is to raise awareness of the importance for us —as scientists in the muscle field and trainees—to be more precise when defining or referring to “fiber types” in our studies. This is critical so we do not inadvertently propagate false assumptions or overlook additional relevant factors as we move forward.

## TRAP 1: ASSUMING THAT CLASSIFICATION BY MYOSIN ISOFORM AND FIBER OXIDATIVE CAPACITY IS EQUIVALENT

The wide range of physical capabilities achievable by mammals and the actions of different muscles within an animal/human are accomplished by the vast heterogeneity of functional properties across skeletal muscle fibers. In this section, we will focus on two qualities: contractile speed and fatiguability, that is the ability to maintain force and/or power over time; however, we also acknowledge that the vast heterogeneity of muscle functional properties are also dictated by variations in muscle architecture (including physiological cross-sectional area, pennation angle, optimal fiber length, sarcomere operating range, and tendon properties). Contractile speed and fatiguability are common and well-recognized parameters that have each been used to classify muscle fibers into types often simply referred to as slow-twitch or fast-twitch muscle, or sometimes red or white muscle. The binning of fiber type into these binary classifications (Table 1) has an historical basis, but has also created the temptation to assume that these classification schemes map onto one another. This is simply not so, as the mechanistic bases for contractile speed and fatiguability are entirely distinct. Moreover, the development of antibodies for specific myosin isoforms has provided a definitive experimental approach for classifying fibers based on myosin heavy chain isoform expression, but it has done little to dispel the misconception that oxidative capacity and fatiguability are also determined by the “slow” versus “fast” classification. It is important to recognize that metabolic properties across muscle fibers constitute a continuum that varies across muscles, species, habitual levels of activity, and disease state and can be and often are disassociated from myosin type.

**Table 1.** Classification schemes

Muscle/Fiber-Type Classifications	Example Measurement	Tissue/Fiber Specific	Representative Reference	Recommended Use	Continuous vs. Discrete	Subjective vs. Objective	Method Complexity
Muscle color	Red/white visual	Tissue	(1)	General descriptor: coloring based on metabolic features, not myosin	Continuous (more red or less red)	Subjective	Low
Myosin ATPase	Myosin enzyme activity measure: visualization of product formed	Fiber	(2)	Myosin typing individual fibers	As an enzyme activity assay, staining intensities are continuous, but it is used discretely	Subjective	Mediump H sensitive; requires extensive optimization by species and tissue
Myosin heavy chain antibody	Detects specific myosin heavy chain proteins	Fiber	(3)	Current best practice for classifying fibers by myosin type	Discrete	Objective	Low
SDH activity: succinate dehydrogenase activity	Mitochondrial enzyme activity assay	Fiber	(4)	Used to delineate variations in oxidative metabolism; localized to mitochondria	Continuous	Subjective	Medium Attention to timing required
NADH-TR activity: reduced nicotinamide adenine dinucleotide-tetrazolium reductase	Mitochondrial enzyme activity assay	Fiber	(5)	Used as a marker of oxidative metabolism; the sarcoplasmic reticulum as well as mitochondria contribute to activity	Continuous	Subjective	Medium Attention to timing required
Muscle fiber size	Use laminin or dystrophin antibodies to outline muscle membrane	Fiber	(6)	Morphological property	Continuous	Objective	High (see Trap 2)
Contraction velocity: maximum velocity of unloaded shortening	Mechanical measurement of speed of shortening, correlated to twitch contractions time during isometric contraction	Muscle	(7)	Functional property	Continuous variable but can bin by speeds	Objective	High Requires specialized equipment; sensitive to conditions such as temperature, isometric versus concentric
Fatiguability	Mechanical measure: rates of force loss during repeated contractions	Muscle	(8)	Functional property	Continuous	Subjective	High Highly dependent on choice of contraction protocol; sensitive to conditions such as temperature, in vivo versus in vitro, electrical stimulation versus voluntary contractions
Specific force	Mechanical measure: maximum isometric force normalized for physiological cross section	Muscle or fiber	(9)	Functional property	Continuous	Objective	High (see Trap 3)

### Historical Context

Identification and analysis of fiber types has a long history going back ∼200 years. The initial distinction was through recognition of different tissue colors—red versus white—and this has evolved over time to include molecular, biochemical, and biophysical measures to distinguish populations of fibers whose divergent properties contribute to muscle function. Fiber typing was originally based solely on metabolic properties, which were inferred from oxidative enzyme activities determined through enzyme histochemistry performed on muscle cross-sections. In particular, the use of succinate dehydrogenase (SDH) and/or nicotinamide adenine dinucleotide dehydrogenase-tetrazolium reductase (NADH-TR) activities as features to distinguish muscle fiber types came into wide usage in the 1960s (10–14). SDH is a key enzyme of the Krebs cycle located at the inner mitochondrial membrane and NADH-TR is similarly an oxidative enzyme, and thus both are frequently used as surrogates for mitochondrial content and oxidative capacity in muscle fibers. Although these techniques became widely used, even early on there was appreciation of the arbitrary nature of using histochemical stains for enzyme activities to delineate fiber types due to the subjectivity in distinguishing high, intermediate, and low levels of staining. In Table 1, we list several commonly used fiber classification methods and note which are subjective in nature and subject to methodological complexities. Moreover, without measures of muscle function or contractile properties to accompany the histochemistry, the limited significance of these classification schemes was recognized (2).

Links between oxidative capacity and muscle function were initially demonstrated by experiments that found motor unit fatiguability was correlated with SDH activity (15). In these classic experiments, individual motor axons were repetitively stimulated while measuring maintenance of muscle force over time to identify motor units that were either fatigue resistant or highly fatiguable. After motor unit force measurements were completed, serial sections were stained for glycogen depletion to identify the fibers contained in the contracted motor unit. SDH staining of these same fibers was used to identify whether the fibers had high or low oxidative capacity. In this way, SDH-positive or -negative fibers could be related to their contractile force characteristics. These studies established the clear presence of SDH-positive or SDH-negative fibers in motor units that either maintained force well over time (e.g., fatigue resistant) or showed a rapid decline in force (e.g., fatiguable), respectively (15). Although the experiments of Edstrom and Kugelberg (15) demonstrated an association between fatiguability and oxidative capacity, they also clearly showed that motor units with fast contractile speed did not universally display the characteristics of high fatiguability and low SDH activity. In fact, fast motor units often showed high resistance to fatigue as well as strongly SDH-positive fibers. Finally, motor units that exhibited a wide spectrum of intermediate SDH staining and a range of fatigue resistance were also identified. Although these very early experiments provided insight into metabolic and muscle function associations, they also unmistakably indicated that metabolic properties were variable and fibers with high metabolic enzyme levels are not restricted to those muscles containing fibers with slow maximum shortening velocities. Thus, the oversimplification of a one-to-one relationship between metabolic capacity and contractile speed was not in general supported by the findings from this paper.

During roughly the same period as the experiments described in the previous paragraph, Bárány (16) used biochemical methods to isolate and assess the ATPase activities of myosin from 14 different muscles obtained from mammals, other vertebrates, and even invertebrates with known and widely varying speeds of shortening. The results of this work demonstrated that the ATPase activity of the myosins was inversely proportional to the contraction time of the muscles; i.e., the higher the ATPase activity, the shorter the contraction time (faster contraction). The correlation between myosin ATPase enzyme activity and contraction speed motivated the development of enzyme histochemical procedures to identify myosin ATPase activities. The discovery that the pH stability of the myosin ATPases varies (17) allowed investigators to apply this biochemistry to muscles in cross-sections (18). Exposing frozen sections to an acid or alkali pH before staining for ATPase resulted in the ability to distinguish two populations of fibers; high-ATPase/fast contracting fibers were revealed to be alkali-stable and acid-labile, whereas low ATPase/slowly contracting fibers were acid-stable and alkali-labile.

### Links between Oxidative Capacity and ATPase Activity

The development of enzyme histochemical procedures to identify myosin ATPase activities allowed for the simultaneous experimental mapping of contractile properties and metabolic properties using motor unit contractility, SDH levels, and myosin ATPase activity. Using this approach, Burke et al. (8) identified three nonoverlapping “types” of motor units in cat gastrocnemius muscle for which physiological properties could be approximately matched to histochemical fiber types. These elegant experiments created an additional layer of complexity to the classification of motor units based largely on function with the emergence of slow (S), fast fatigue resistant (FR), and fast fatiguable (FF) as motor unit “types” (8). Motor units with fast contraction times and high fatiguability (FF motor units) were composed of muscle fibers that stained weakly for SDH but showed high myosin ATPase activity, muscle fibers in fast motor units with the ability to maintain force over time (FR motor units) similarly had high myosin ATPase activity but also high SDH activity, and motor units with slow contraction times were composed of muscle fibers that displayed very low myosin ATPase activity and high SDH activity. These findings created the basis for a simple classification scheme categorizing muscle fibers by both contraction characteristics and metabolic properties.

Although the findings from Burke et al. (8) provided a clear motor unit classification system for the cat gastrocnemius muscle, other studies emerged that demonstrated the ability to reliably differentiate only two fiber types by assaying myosin ATPase activity at alkali pH (4), whereas others (2) reported muscle-specific, as well as species-specific, differences in the pH sensitivities of myosin ATPase activity. A comprehensive evaluation of multiple enzymatic and substrate characteristics of numerous muscles from both guinea pigs and rabbits (19) proposed a three-category classification system similar to that of Burke et al. (8), termed fast-twitch-glycolytic (FG), fast-twitch-oxidative-glycolytic (FOG), and slow-twitch-oxidative (SO) that was purposefully left somewhat open-ended as to allow a nomenclature that can accommodate other potential permutations and combinations of contractile and metabolic patterns, e.g., fast-twitch-oxidative or slow-twitch-glycolytic, which were actually expected in various species or under different physiological conditions, such as following exercise training (19). In fact, Nemeth et al. (20) subsequently demonstrated significant heterogeneity of metabolic properties of fibers classified broadly into either two or three groups by myosin ATPase. By this time, another nomenclature for classifying fiber types had emerged: “Type I” fibers having low ATPase activity, “Type IIA” having moderate ATPase activity, and “Type IIB” having high ATPase activity (2), whereas Nemeth et al. (20) showed that Type II fibers displayed the entire range of oxidative enzyme activities, whereas some Type I fibers were identified that contained high levels of glycolytic enzymes. Thus, collectively the studies of fiber-type classification performed throughout the 1970s clearly revealed that muscle fiber oxidative capacity cannot be conclusively inferred from myosin ATPase activity, and conversely, contractile speed or myosin ATPase activity cannot be predicted from oxidative enzyme activity.

Continued reference to these incorrect assumptions in the literature is surprising in light of the observation made by Brooke and Kaiser (2) in the opening of the summary section from their paper over 50 years ago that the “nomenclature of muscle fiber types is beset by problems” and they pointed out that “classification of striated muscle into different types has always been somewhat confusing but recently has shown an alarming trend toward the incomprehensible.” The difficulty is inherent in ascribing the entire range of potential physiological functions to discrete contractile and metabolic properties, especially when even the very early studies showed that in many cases the markers of physiological function that were used failed to show unambiguous associations. Brooke and Kaiser were significantly ahead of their time in their conclusion that any system of muscle fiber classification should *1*) be based on the specific fiber properties (e.g., contractile speed) being examined, *2*) have practical application to experimental or pathological situations, and *3*) allow clear-cut differentiation of fiber types without any gradual transition from one fiber type to another or many indeterminate fibers. They also quite aptly pointed out the importance of resisting the temptation to assume that different classification schemes could be equated. These constraints have been largely addressed for one muscle function parameter—contraction speed—by the development of antibodies directed toward specific fast and slow myosin heavy chains (MyHC).

### MyHC Isoforms Are the Best Available Marker for Fiber Typing but Provide No A Priori Information about Metabolic Properties

In the late 1980s, monoclonal antibodies were developed against the various myosin heavy chain (MyHC) protein isoforms. In addition to the MyHC type I, type IIA, and IIB, a fourth isoform termed IIX was identified (3). Fibers expressing MyHC type IIX were widely distributed in rat skeletal muscles and could be distinguished from fibers expressing MyHC type IIA or IIB both by the lack of reactivity with the IIA or IIB antibodies as well as by myosin ATPase histochemistry (3). At roughly the same time, a group using precise microdissection techniques of single muscle fibers and electrophoretic procedures with improved sensitivity also identified four MyHCs (21, 22). The fourth isoform was termed MyHC type IId leading to some transient confusion in the field, until it was established that MyHC IId and the aforementioned MyHC type IIX were in fact the same protein from the same gene. In general, the muscle field uses the term IIX for these fibers. Most importantly, work in human muscle determined that fibers previously classified as type IIB by myosin ATPase staining were actually expressing MyHC type IIX (23), and MyHC type IIB is now recognized to be rarely, if ever, expressed in human muscle. The availability of myosin-specific antibodies has also increased our awareness that myosin isoform expression does not predict metabolic capacity of the fibers. Specifically, fibers in human muscles expressing MyHC type IIX exhibit very low levels of SDH activity (24), whereas MyHC type IIX fibers have moderate SDH staining in rats (3, 25) and high SDH activity in mice (26, 27) despite displaying high ATPase activity across the board.

Thus, using immunofluorescent staining with specific antibodies for MyHC isoforms is the most definitive means of identifying and classifying muscle fibers, but the primary inference from this analysis is related to contraction speed. This method of classification meets the requirements suggested by Brooke and Kaiser in 1970, but it must be recognized that it provides no information by itself regarding the metabolic properties of the fibers. Secondary methods, including staining for oxidative enzymes, such as SDH, are required to glean information regarding oxidative capacity. Although the use of antibodies to classify fibers is a definitive and straightforward approach, it should also be recognized that under conditions of increased or decreased loading, increased or decreased neuromuscular activity, aging, or disease, fibers frequently coexpress multiple MyHC isoforms (28, 29). The presence of so-called hybrid fibers that coexpress two or more MyHC isoforms is posited to be an indication of a fiber undergoing a switch in fiber type (30). A final point to stress is that fiber typing is typically performed on a few cross-sections representing only a tiny portion of the length of the muscle fibers. In general, it is assumed that the contractile and metabolic properties are uniform along the length of the fiber, but there can be regional differences in oxidative enzyme activities along fiber lengths (31). Finally, muscle fibers can unquestionably display dramatic alterations in metabolic properties under conditions that do not elicit changes in MyHC composition (32).

## TRAP 2: ASSUMING THAT FIBER CROSS-SECTIONAL AREA IS A SURROGATE MARKER FOR MYOSIN ISOFORM OR OXIDATIVE CAPACITY

Another characteristic of muscle fibers that is often assumed to be related to myosin isoform or oxidative capacity is fiber cross-sectional area. It is commonly stated that “fast” fibers have larger cross-sectional areas as compared with “slow” fibers. One can certainly find specific studies reporting that muscle fibers with fast myosin isoforms were on average larger than fibers with slow myosin isoforms; however, this is not a generalizable finding due to multiple factors, including the substantial heterogeneity in fiber cross-sectional area within muscles, the substantial differences in myosin type distribution between muscles, the differences that exist between species, significant differences between males and females, and changes during the life span. Making assumptions regarding the relationship between fiber size and myosin isoform or metabolic characteristics in designing experiments, interpreting data, and building models could lead to misleading or incorrect conclusions.

### Historical Context

Initial work classifying fibers by enzyme activities led to hypotheses about fiber size based on oxidative versus glycolytic capacity (33, 34). These initial studies that cataloged select muscles in rats found that fast fibers were larger than slow fibers. This conclusion did not seem unreasonable, as it was consistent with the notion that lower cross-sectional areas for oxidative fibers would achieve higher surface-to-volume ratios compared with glycolytic fibers and thereby facilitate diffusion of oxygen within the fiber (8). Moreover, it would theoretically be more energetically efficient for fast glycolytic fibers to have a lower surface-to-volume ratio (and thus have higher cross-sectional areas) so they could generate high forces yet require less energy to maintain the fiber membrane machinery (associated with maintaining ion gradients and membrane potential). These concepts were explored in a highly influential paper by Armstrong and Phelps (33) in which muscle fiber type and cross-sectional areas were carefully catalogued for hindlimb muscles of six male Sprague-Dawley rats. Armstrong and Phelps used NADH-TR activity as a marker of mitochondrial oxidative capacity and myosin ATPase activity to delineate myosin types. Combining these histochemical methods allowed the authors to identify fast-twitch glycolytic (FG), fast-twitch oxidative-glycolytic (FOG) and slow-twitch oxidative (SO) fibers, based on the methods described in the previous section (19). The histological sections were then analyzed to calculate fiber cross-sectional area for each fiber type by manually digitizing the images. Consistent with the surface-area theory, the findings of Armstrong and Phelps (33) demonstrated a general trend that the average cross-sectional areas of the FOG and FG fibers were higher than those of the SO fibers. However, there were minimal to no differences in the cross-sectional areas between FOG and FG fibers notwithstanding dramatic differences in oxidative capacity. The authors acknowledged the “surface area” theory and stated that their findings were generally supportive of it, though they cautioned the readers about overgeneralization of their results. These results were reinforced by the study of Delp and Duan (34) in which fibers were quantified similar to Armstrong and Phelps; NADH-TR activity was a marker of mitochondrial oxidative capacity and myosin ATPase activity delineated myosin types. Although their analysis yielded smaller average cross-sectional areas for the fibers expressing MyHC type I than those expressing type IID/X and IIB MyHCs across a wide range of forelimb, trunk, and hindlimb muscles, the study included data from only three male Sprague-Dawley rats. Moreover, the authors noted several exceptions to the surface-area theory and also acknowledged the fact that fiber cross-sectional area is highly dynamic and changes due to varying loading conditions.

Despite the inconsistencies across muscles and the fact that collectively these two studies (33, 34) analyzed a total of eight rats of one sex and a single strain, the findings are highly cited as evidence for generalized statements relating myosin isoform and metabolic classification and fiber size. It must be acknowledged that these studies were exceedingly labor intensive, very carefully done, and the conclusions contained within each paper entirely valid, but to generalize this concept across muscles and across species is not justified. In fact, Delp and Duan quite appropriately stated in their paper: “Muscle fiber size, composition, and oxidative potential reported herein may only be applicable for rats of the same strain, age, and gender.” As they anticipated, there are many findings in the literature that are contrary to those of Delp and Duan, as discussed in the next paragraph.

### Fiber Cross-Sectional Areas Are Highly Muscle, Species, and Sex Dependent

Experiments performed in mice and humans have clearly illustrated that the general trend of fast fibers being larger than slow fibers does not universally hold across muscles, species, or between sexes. For example, contrary to the generalization put forth by the previously discussed rat data, Augusto et al. (35), using the myosin ATPase technique, found that while in the EDL muscle of C57BL/6 mice, fibers with low ATPase activity (“Type I”) were on average smaller than fibers with high ATPase activity (“Type IIA, IIAD, and IID”), this finding did not hold true for other muscles. In the soleus, Type 1 fibers were larger than Type IIA, IIAD, and IID. And, in the gastrocnemius muscle, Type 1 fibers were larger than Type IIA and IIAD but smaller than Type IID. Clearly, the relationship between fiber size and type found in the early rat studies is not the same in mice, and it is even different across muscles within the same species. Furthermore, the literature describing fiber cross-sectional area and fiber type in humans is highly variable. Although some of the variability in the human data may be due to differences in biopsy sites (36), the data certainly do not support a generalized relationship between fiber size and fiber type for human muscle, especially when incorporating both male and female datasets (37–39). Indeed, there are significant differences between males and females in terms of the cross-sectional areas of each fiber type within a given muscle. For example, Jeon et al. (38) used gel electrophoresis to distinguish MyHC I, IIA, and IIX in human vastus lateralis biopsy samples and found that while in males, MyHC IIA fibers were bigger than I and IIX, in females, MyHC I fibers were the biggest. It is important to note that most of these studies have somewhat limited study population sizes, and given the diversity in genetics, environment, and age in the populations, a finding from a handful of studies should not be considered universal; however, a generalization that fast fibers are larger than slow fibers is certainly not supported by the existing data.

### Advanced Histological Analyses Reveal That Cross-Sectional Areas Vary Substantially Throughout Muscles and within Fiber Types

**Figure 1.**
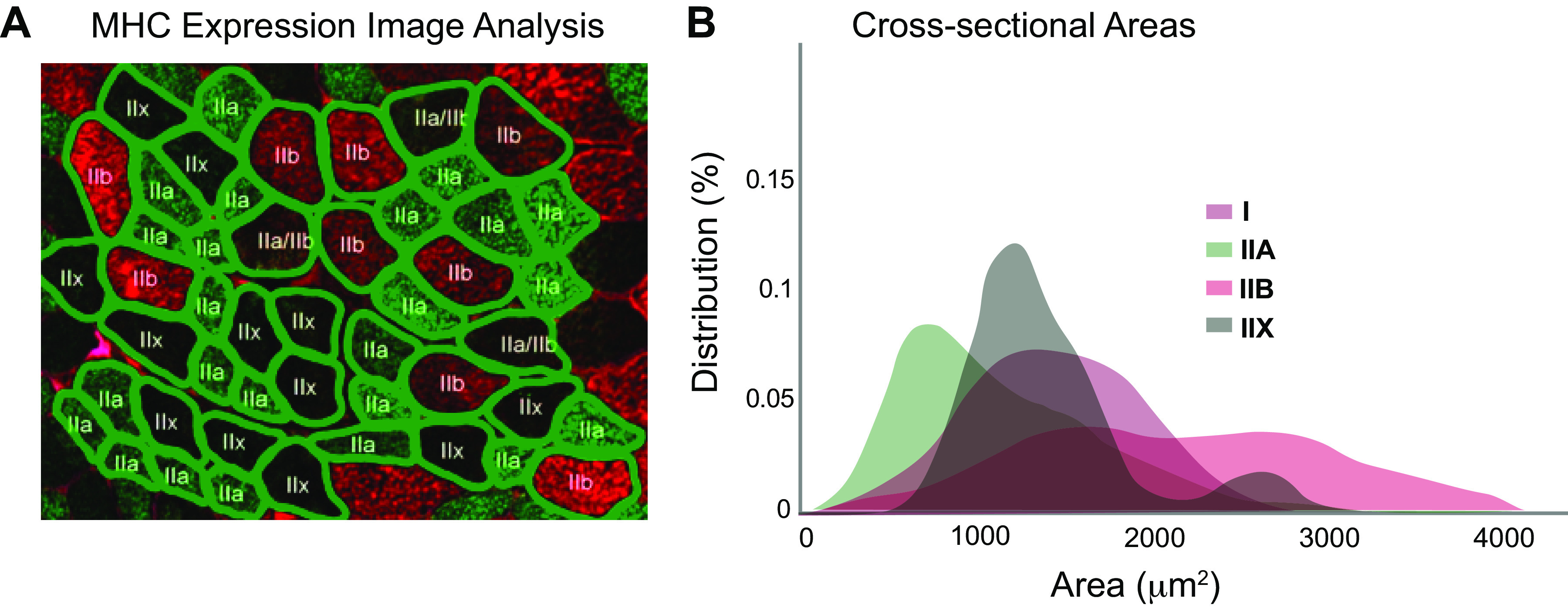
Illustration of segmentation of ∼50 muscle fibers (*A*) from one muscle section stained for myosin heavy chain (MHC). An automated method is used to detect fiber boundaries, and fiber types are labeled automatically based on color. This analysis leads to measurement of variation in cross-sectional area across fibers (*B*), clearly showing significant overlap in size across fiber types. Adapted from Liu et al. (41).

Advanced image processing approaches that automatically detect fiber boundaries (40–43) have enabled researchers to measure fiber cross-sectional areas throughout the entirety of muscle sections, whether obtained through biopsies of human muscles or through sectioning of whole muscles extracted from animal models. These studies have made it clear that fiber CSAs vary substantially throughout muscles, and therefore extrapolating from the average value for muscle fiber CSA is not representative since the distributions in fiber sizes can vary substantially yet reveal no difference in the overall average. Furthermore, when CSAs are measured for fibers labeled for MyHC isoform, analysis of the fiber-type specific CSAs shows substantial overlap in the distribution of fiber sizes, even when the average CSAs differ between fiber types (Fig. 1). For example, in the study by Encarnacion-Rivera et al. (40), automatic analysis of cross-sectional images of MyHC florescent images allowed for characterization of full distributions of gastrocnemius muscle fiber areas, demonstrating significant overlap in cross-sectional areas among fibers expressing MyHC I, IIA, IIB, and IIX in wild-type mice. Similarly, quantification of biopsies taken from human vastus lateralis muscles demonstrated wide variation in fiber CSA of Type I and Type II fibers (identified with MyHC antibody labeling), with significant overlap between the types (44). Furthermore, in some muscles, there appears to be specific differences in fiber type and CSA between superficial and deep portions of muscles (45), which creates further challenges associated with localized measurements from biopsies since they selectively sample superficial regions. The wide variation in CSAs and the significant overlap between the CSAs of fibers with varying myosin isoforms further illustrate why assuming that there is a generalizable relationship between CSA and myosin isoform is incorrect and could lead to misinterpreting data.

### Fiber Cross-Sectional Area and Oxidative Capacity Are Highly Adaptable

The last several decades have produced a wealth of information demonstrating that muscle fiber cross-sectional area is highly adaptable and responds to changes in use and mechanical stimuli (46, 47), resulting in changes in cross-sectional areas of the fibers that can and do differ across fibers that express different myosin isoforms. When the habitual level of load placed on a muscle increases, such as through resistance exercise, muscle fiber cross-sectional area increases by the addition of myofibrils in parallel, whereas with disuse or immobilization there is a corresponding decrease in the number of myofibrils in parallel (46). Although all fiber types respond to chronic changes in loading, fiber-type-specific responses may vary, even in response to the same altered loading environment (48). Fiber areas are also known to be highly affected by muscle disease, age, and neurological injury (49). Thus, many confounding factors likely play a more important role in relative fiber cross-sectional areas than does fiber type, per se.

### Indirect Approaches That Assume Fiber Area Is Related To Fiber Type Should Be Used with Caution

With relevance to clinical as well as translational studies, the ability to measure muscle fiber characteristics and “type” noninvasively in humans is desirable, and fiber morphology is much easier to capture than shortening or metabolic behavior using medical imaging. Thus, cross-sectional area as a surrogate for fiber-type classifications has been appealing. For example, a magnetic resonance imaging (MRI) based approach called diffusion tensor imaging (DTI) has become a commonly used tool in the muscle mechanics community because it allows for characterization of the complex three-dimensional arrangement and properties of muscle fascicles and fibers within muscle (50, 51) leveraging diffusion characteristics of the muscle fibers. Indeed, there is evidence that the speed of diffusion measured with DTI is related to the cross-sectional area of the muscle fibers as measured from histological samples taken from muscle biopsies (52). However, given the caveats listed earlier, this should not be taken as a surrogate for fiber myosin isoform or oxidative capacity, although some have explored using it in this way. Sheel et al. (53) reported that the human tibialis anterior and soleus muscles have both differing diffusion and fiber-type distribution characteristics in 12 healthy males, but as in other studies, this finding cannot be extrapolated. Instead, there is the promise of other imaging approaches that attempt to image for features thought to be correlated with myosin isoform and/or oxidative capacity, including transverse magnetization (T2) relaxation times (54) quantified by MRI, muscle carnesine imaging by ^1^H-MRS (55), and q-space MR imaging (56). These efforts provide exciting hope for future in vivo, noninvasive options for assessing fiber-type composition with imaging, but further investigation is required to evaluate the generalizability of these techniques across muscles, individuals, and species.

## TRAP 3: ASSUMING THAT MUSCLE FORCE-GENERATING CAPACITY CAN BE INFERRED FROM MYOSIN ISOFORM

A third characteristic that is often associated with fiber type is force-generating capacity. Would a muscle that comprises more Type II fibers necessarily generate a higher peak isometric force per unit area than a muscle with Type I fibers? This is actually not a simple question because the wide range of approaches used to measure force and cross-sectional area across length scales yield highly varying results. Further complexity is introduced because scientists that study muscle force come from a range of backgrounds—including biology, physiology, biophysics, movement sciences, and engineering—and the lack of consistency in terminologies across those fields introduces additional confusion.

### Historical Context

Dating back to the earliest reports of muscle strength measures, researchers recognized that to make meaningful comparisons regarding force-generating capacity across muscles and across scales, it is necessary to normalize force generation by muscle cross-sectional area (57). Such normalization, which is termed “specific tension” (or “specific force”), controls for differences in the mass or volume of contractile material in the muscle (or motor unit or fiber) being examined. Specific tension is also sometimes referred to as peak isometric muscle stress and has the units of force per unit area (e.g., Newtons/cm^2^). The terminology is similar to other normalized parameters in chemistry and physics (e.g., specific heat) in which a characteristic of a given substance is expressed as the ratio of the measure normalized by either the mass of the material or by the measure in a standard substance such as water. Not only is specific tension a useful parameter that allows for comparison across muscles of different sizes or from different animals or anatomical locations, and similarly from fiber to whole muscle level, it is also a quantity that shows some consistency across mammalian skeletal muscle (at the fiber level and at the tissue level if muscle architecture is considered) and thereby serves as a conserved marker for healthy skeletal muscle (58). This consistency is supported by specific force measures for muscles across a wide size range of mammals (0.1 to 300 kg) (59, 60). However, despite the clear importance of using a normalized force value to appropriately examine force-generating capacity, not all investigators use sufficient care or precision with units or language when reporting force measurements. For example, some investigators may use the term “tension” or “force” without “specific” as a modifier, which implies an unnormalized force value even if values are reported in units of stress. At the whole limb level, there is often casual and interchangeable use of terms such as strength, force, torque, or moment, and in some cases, values are described inconsistently with reported units. Such inconsistency can lead to confusion and difficulty in moving fluently across studies and fields.

Although a general conservation of a “typical” specific force value is widely accepted, other work suggests there may be fiber type-specific differences in force generation. To examine this question, one must recognize the widely varying methods used for determining muscle force and cross-sectional area and the extent to which these methodological details may contribute to differences due to technical rather than biological differences (see Table 1). For example, depending on the scale considered, the measured cross-sectional area may indeed include only myofibril density and structures such as mitochondria and sarcoplasmic reticulum (SR); at larger scales, the measured area may include other noncontractile elements such as fat or fibrosis, especially at the whole muscle level (61). Here, we examine the evidence for type-specific force-generating capacity across a range of length scales, and the challenges in data interpretation arising from methodology and scale.

### Relationship between Force and Myosin Isoform at the Protein and Single Fiber Levels

**Figure 2.**
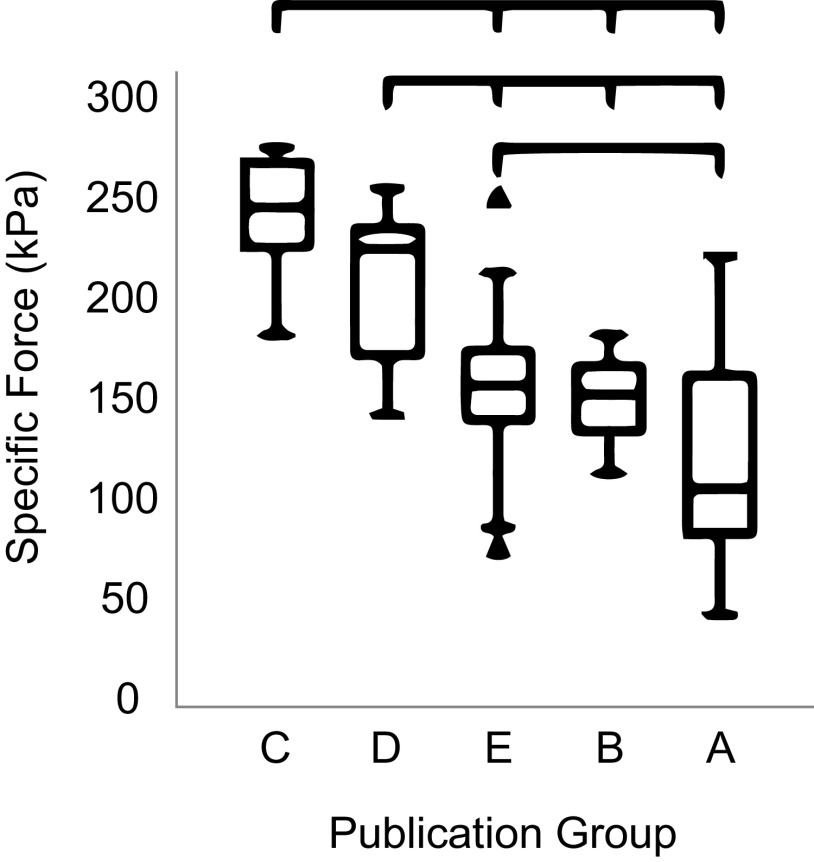
Meta-analysis revealing striking differences in specific force measured from the same fiber type in the same species between different lab groups. Adapted from Kalakoutis et al. (62).

The most conclusive means of relating myosin isoform and specific tension is through experiments in which force of single isolated muscle fibers is measured while controlling length and activation level and with subsequent direct evaluation of the constituent myosin isoforms, or via direct measures of cross-bridge force generation. Even for these definitive and well-controlled studies, wide variation appears in the literature in reported force and specific tension values. A meta-analysis that considered 61 studies of human muscle fibers showed a many-fold variation in specific forces reported in the literature even for a single myosin isoform type derived from young healthy adults and analyzed at the same temperature (62). That same analysis showed higher variation between research groups than between fiber types (Fig. 2); however, the data did confirm that fibers expressing MyHC Type IIa consistently generated higher specific forces than MyHC Type I fibers, with the difference on the order of ∼24% (62). Data from other species have also reported fiber type differences for specific force. For example, in dogfish (63), white muscle fibers were reported to have a higher specific tension (289 kPa) than red fibers (142 kPa). However, there are instances in which the converse is observed. In muscles of the chicken (64), fast fibers of the posterior latissimus dorsi were reported to have a similar or lower specific tension (100 kPa) than fibers of the slow anterior latissimus dorsi (100–150 kPa) [muscle type characterized by twitch velocity and force duration (64) in the presence of potassium-rich solution]. Work by Lucas et al. (65) reported no significant differences in specific tension between muscle fiber types in the cat medial gastrocnemius, with fiber type assessed using actomyosin ATPase histochemical assays. Overall, comparisons between experiments, especially between different research groups, are highly problematic, but when direct comparisons are made between different fiber types, the collective findings indicate that at the single fiber level, fast fibers (i.e., fibers expressing MyHC Type II) generate moderately higher specific forces than slow fibers expressing MyHC Type I.

One possible explanation for fiber type-specific differences in force generation is a difference in the density of contractile proteins within the fibers. If slow fibers have a higher density of mitochondria, as is generally accepted and reported by Bone et al. (66) for white and red fibers of dogfish, the density of myofibrils and force-generating structures would necessarily be lower, which could theoretically account for relatively lower specific tension. Lou et al. (63) considered this possibility and adjusted their specific tension values by taking account of the volume occupied by nonforce-generating components. With this correction, the fiber-type differences persisted with adjusted specific tension values still more than 50% greater for white compared with red fibers. Therefore, myofibrillar density is not likely the major factor accounting for fiber-type differences in specific tension at the single fiber level.

An alternate possibility is that different MyHC isoforms have inherently different force-generating capacities. Schiaffino and Reggiani (67) suggested that the potentially higher force generation in fast MyHC isoforms may arise from more cross-bridges exerting a higher force per cross bridge, whereas others (68) assert that force per cross-bridge is approximately equivalent between myosin types and the force difference derives only from the number of cross-bridges. Geiger et al. (69) explored this question in isolated muscle fibers from rat diaphragm and determined both myosin content and myosin isoform composition using Western blot analysis. Their findings showed that fibers with IIX or IIB/IIX isoforms had MyHC content threefold higher than in those expressing the IIA MyHC isoform. When specific tensions were corrected by MyHC content, values for slow fibers of 0.7 N/μg MyHC and for fast fibers of 1.2 N/μg MyHC suggested that fast myosin isoforms were inherently stronger than slow isoforms. In vitro motility assays are generally consistent with this observation, reporting that the force-generating capacity of Type I myosin is lower than that of Type II myosin isoforms, with no detectable differences between MyHC Type IIA and Type IIX (70). In sum, although there is evidence that at the cross-bridge level fast MyHC produces higher forces than slow MyHC, it is still a controversial subject and the manifestation of any potential differences in MyHC force production at the fiber level is debatable.

### Additional Challenges Associated with Methodological Differences

Challenges in interpretation and comparisons across studies to reach a consensus on this have arisen in part from differences in experimental approach. Temperature, composition of the media, and method of skinning or permeabilizing among other factors affect the magnitude of forces measured, whereas methods of estimating area or accounting for swelling can further impact specific tension calculations (62). Specific tension is highly temperature dependent, with more sensitivity exhibited at temperatures in the range of 12–17°C but less so in higher temperatures up to 22°C (71). Comparisons of various chemical skinning and storage methods revealed effects on specific force values but not shortening velocity or cross-sectional area (72). Some individual studies have used different approaches to address the fiber swelling that occurs upon chemical skinning. Use of osmotic compression to reverse fiber swelling indicates that fiber swelling reduces calcium sensitivity of the contractile proteins in Type II fibers (typed based on Sr^2+^ sensitivity) (73, 74) and force generation in soleus fibers (described as slow based on contractile speed) (75). Because of the dependence of force on velocity, how velocity is controlled in a given experiment is also an important consideration. In one study (76), larger force depression was observed in Type IId than in Type I fibers (defined by MyHC content) when fiber length was shortened at the same absolute speed in fiber lengths per second, but no differences when the prescribed shortening speed was defined relative to the fiber’s maximum shortening velocity. Because of the force-length dependence of muscle force generation, it is clear that fiber length must also be controlled when evaluating specific tension. However, while some studies carefully constrain fibers to their optimal fiber length when measuring the force generated [such as in rat diaphragm (69) and in dogfish (63)], others record force generated with an intact fiber with the limb in a fixed posture, such as (64) in chickens (limb fixed with joints in 90 degrees). In the latter conditions, optimal fiber length, and thus peak specific tension, is not assured.

### Relationship between Force and Fiber Type at the Whole Muscle Level

Establishing the existence of fiber type differences in force-generating behavior at the whole muscle level is confounded by numerous issues related to muscle fiber-type composition, fiber orientation and architecture, and differences in noncontractile elements. These factors and others interact in complex ways to create functional behavior of a given muscle (77). Whole muscle behavior is nevertheless often extrapolated from single fiber work, akin to asking whether a whole muscle exclusively of a single fiber type would have the same force as another of the same mass and architecture with a different fiber type. Rather than approaching the question of whole muscle-specific tension from the summation of individual fibers, whole muscle-specific tension estimates are also generated using a variety of approaches including inverse dynamics, which requires isometric or isokinetic maximal torque information, estimates of muscle-tendon moment arms, and muscle architectural information. In animal experiments, these critical characteristics can be assessed using postmortem dissection and fiber-type determination (59), but most human whole muscle studies require assumptions from cadaveric measures of muscle architecture to be applied to in vivo measures of force and torque production (78). More recent approaches attempt to directly estimate muscle volume, length, and moment arm using CT (79), dual-energy X-ray (80), ultrasound (81), or MRI (82–86), although none of these methods are suitable for measuring optimal fiber length nor fiber type.

Given this complexity, it naturally follows that there are discrepancies between fiber and whole muscle measures. For example, one meta-analysis (87) examined whole muscle and individual fiber characteristics and specific tension following resistance training. The authors reported no differences in hypertrophy at the fiber and whole muscle level, yet strength gains were significantly larger for the whole muscle relative to the individual fibers, perhaps due to changes in motor unit recruitment. Moreover, a detailed evaluation of maximum voluntary strength and muscle cross-sectional area and fiber-type composition revealed no correlation between specific force at the whole muscle level with percent Type I fiber area as determined by myosin ATPase histochemistry (88). In contrast, another study (89) reported significantly larger (2 to 3 times) specific tensions for fibers with high compared with low ATPase activity on the basis of overall quadriceps force relative to fiber type areas as determined from whole muscle forces measured in vivo. Based on the modest differences in force-generating capacity observed at the level of single fibers, other factors such as complex architecture, musculoskeletal geometry, coordination, motor unit recruitment, and force transmission may play a more important role than fiber type in determining muscle tissue level force generation, especially after training and following injury or impairment.

## DISCUSSION

**Figure 3.**
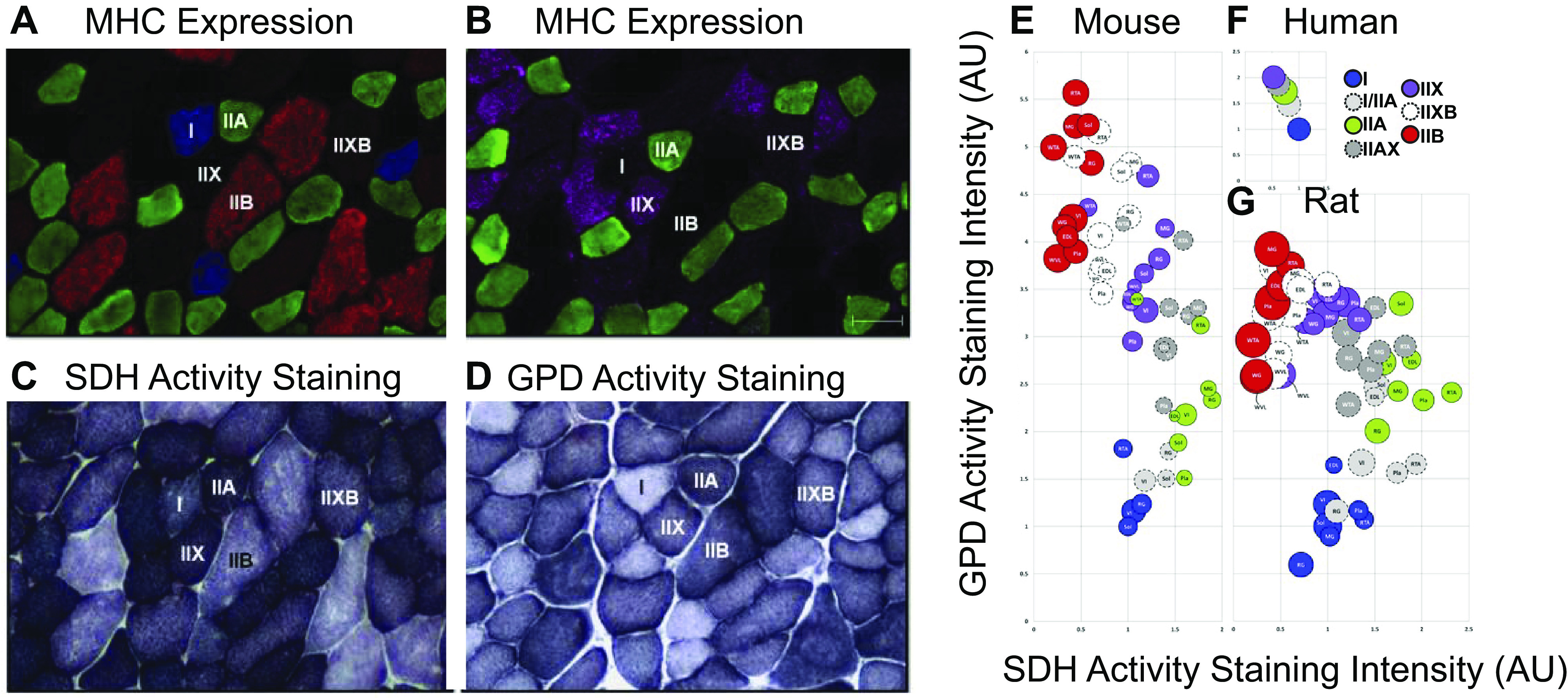
Representative images of mouse gastrocnemius muscle showing myosin heavy chain (MHC) expression (*A* and *B*) as well as succinate dehydrogenase (SDH) activity staining (*C*) and glyceraldehyde-3-phosphate dehydrogenase activity (GPD) staining (*D*). Bubble plots (*E–G*) show SDH activity, GPD activity, and cross-sectional area (CSA) for each fiber type in mouse, rat, and human skeletal muscles. Bubble size represents the relative CSA within a species. SDH and GPD activity are expressed relative to the values obtained in type I fibers (soleus for mouse and rat, vastus lateralis for human) and assigned a reference value of 1.0. There are significant variations and overlap between characteristics of fibers across experiments, species, and muscles. Adapted from Bloemberg et al. (90).

The goal of this perspective paper is to emphasize foundational work that led to our current understanding of fiber types while also bringing to light three key “traps” regarding commonly held assumptions about fiber types. These traps arose largely from issues such as failing to move forward with new levels of biological understanding, overgeneralizing findings, taking results out of context, and lack of precision in terminology. Acknowledging where these traps came from is important, but we also stress that we are not discrediting the original work that formed the basis for understanding of fiber types and characteristics. Many decades of careful research have formed the basis for our modern and advanced understanding of muscle physiology. However, as the muscle physiology field has advanced, investigators have more interdisciplinarity, and tools have improved, we are suggesting that it is time to step back and to evaluate the large body of data in the literature to refine our conceptual framework for fiber types. So, how do we avoid falling into the traps? We suggest that, as a field, we:

- Recognize the context of our experiments. As discussed extensively in this paper, the context of an experiment that presents fiber characteristics should always be considered when interpreting findings and then applying those to new questions. Examples of context include fiber typing method (myosin, SDH, ATPase, staining protocols, etc.), experimental conditions for functional measures (temperature, bath composition, etc.), species/strain (various mouse strains, rat, human, chicken, fish, pig, dog, cat, frog, horse, worm, mollusk, fly, etc.), the muscle examined, sex (only male, only female, grouped, not grouped, etc.), scale (myofibril, fiber, fiber bundle, whole muscle, multiple muscles), and sample size. All of these contexts have been shown to influence fiber characteristics and outcome measures (Fig. 3) and thus need to be considered and acknowledged before generalizing any findings to new questions.
- Use precise vocabulary across fields. When characterizing muscles in terms of their fiber characteristics and force-generating properties, it is important to be very clear about vocabulary. When describing fiber “types,” we suggest being clear about which characteristics are being measured to classify fibers, whether it be myosin heavy chain isoform, oxidative capacity, ATPase activity, mitochondrial content, etc. Furthermore, when performing measurements on fibers, clarity on the force measurement and normalization method is extremely important. Clarity in vocabulary and definitions will help communication across fields and assure that findings are not misinterpreted or overinterpreted.
- Be clear about what is being measured. Especially with current trends in biomedical science to find the best “biomarkers” for clinical conditions, it is natural to test ideas for ways to find surrogate measures for things like fiber characteristics. However, be mindful of falling into the trap of assuming that the surrogate measure is the same as the actual measure. We present several examples of this issue, including myosin heavy chain versus ATPase activity and myosin type versus cross-sectional area. In addition, given the first bullet aforementioned, be mindful of overgeneralizing findings to develop surrogate measures.
- Understand that modelers and data scientists use data (in absolute form) to inform their work. Advanced computational modeling, systems biology, data science, and artificial intelligence methods are becoming more ubiquitous and as such being applied to key questions in muscle biomechanics, physiology, and function. Therefore, it is important to understand that the data generated experimentally and published in muscle physiology papers are being used to inform, calibrate, and validate those models. These studies will most certainly elevate the impact of muscle physiology studies and advance the field. To facilitate their growth and accuracy, we recommend that experimentalists provide as much data as possible, in particular individual measurements in supplemental data. Furthermore, as the data are discussed, it is important to include explanations of how the absolute values of reported results should be interpreted, keeping in mind that these explanations are not necessarily for the benefit of your close colleagues in the field, but also for those outside the field that are seeking to use the data. Furthermore, for modelers, it is important to read the detailed methods and discussions of these papers to make sure that all the contexts, assumptions, and caveats are interpreted when incorporating the data into the models.
- Acknowledge that fibers exist on a structure-function continuum. Much of the confusion that led to the traps described in this paper stems from the fact that as a field we are trying to classify fibers into bins as opposed to recognizing that they exist on a broad continuum of characteristics. As techniques for characterizing muscle become more advanced and automated, we are becoming more capable of capturing the broader spectrum of fiber characteristics. For example, we will likely glean more information from quantifying fiber areas using area distribution histograms as opposed to extracting one average number for a given sample. Similarly, through advanced nuclear approaches that capture the heterogeneity of transcriptomes within myonuclei of a single fiber can allow us to describe myosin HC expression along a continuum of fiber types, rather than binning fibers in discrete categories of Type 1, IIA, etc.
- Recognize that whole muscle has more complexity than single a fiber. In *Trap 3*, we discuss the various limitations of inferring whole muscle level force generation directly from single fiber force measurements, due to many factors, including muscle architecture, musculoskeletal geometry, coordination, motor unit recruitment, and force transmission. It is important to also acknowledge that the same is true for other muscle characteristics: whole muscle maximum shortening speed cannot be inferred from myosin isoform composition and whole muscle size cannot be predicted from fiber type composition.

In closing, we would like to acknowledge one more “trap” that came to light during our discussions at the 2023 Advances in Skeletal Muscle Biology Conference hosted by the Myology Institute at the University of Florida. The last (or fourth) trap is this: the temptation is great to attribute differences observed in properties (morphological, functional, etc.) between muscles of differing fiber “types” to the fiber type difference. However, there are many differences between muscles, including location, loading history, function, architecture, size, embryological origin, connective tissue structure, and many others. This tendency to focus correlations of physiology or disease outcomes exclusively to fiber type is understandable as it is the most accessible and available marker, but that dominant focus may be limiting our ability to discover additional properties of equal or greater importance. Many decades of research have populated the literature filled with “fiber type” correlations and, to date, relatively few have led to new mechanistic insights. Correlating findings with fiber type is what to some degree led us to these fiber traps to begin with, and it can be particularly problematic if other studies do not find the same relationships. As an example, we heard from some that if their findings do not align or support other correlations with fiber type in the literature, it is dismissed by reviewers as incorrect and unpublishable. This outcome is extremely problematic for our field! Therefore, we encourage the field to move away attempting to correlate all things with myosin type (or other fiber characteristics), given the incredible diversity of characteristics that vary across muscles.

The future of muscle physiology is very bright, with many new tools, techniques, and ideas on the horizon. We encourage the field to avoid falling into these traps with the new techniques and truly let us, and the world, appreciate the breadth of biological, physiological, and functional heterogeneity across muscles!

## GRANTS

This study was supported by Eunice Kennedy Shriver National Institute of Child Health and Human Development (NICHD) under Grant R01HD101406 (to K. R. Saul), National Institute of Arthritis and Musculoskeletal and Skin Diseases (NIAMS) under Grants R01AR079220 (to K. A. Esser), P30AR069620 (to S. V. Brooks), R01AR078396 (to S. S. Blemker), R21AR080415 (to S. S. Blemker), and National Institute on Aging (NIA) under Grant U01AG055137 (to K. A. Esser).

## DISCLOSURES

No conflicts of interest, financial or otherwise, are declared by the authors.

## AUTHOR CONTRIBUTIONS

S.S.B., S.V.B., K.A.E., and K.R.S. conceived and designed research; S.S.B., S.V.B., K.A.E., and K.R.S. prepared figures; S.S.B., S.V.B., K.A.E., and K.R.S. drafted manuscript; S.S.B., S.V.B., K.A.E., and K.R.S. edited and revised manuscript; S.S.B., S.V.B., K.A.E., and K.R.S. approved final version of manuscript.
